# Towards Secure and Privacy-Preserving IoT Enabled Smart Home: Architecture and Experimental Study

**DOI:** 10.3390/s20216131

**Published:** 2020-10-28

**Authors:** Mamun Abu-Tair, Soufiene Djahel, Philip Perry, Bryan Scotney, Unsub Zia, Jorge Martinez Carracedo, Ali Sajjad

**Affiliations:** 1 School of Computing, Ulster University, Belfast BT37 0QB, UK; p.perry@ulster.ac.uk (P.P.); bw.scotney@ulster.ac.uk (B.S.); zia-smu@ulster.ac.uk (U.Z.); j.martinez-carracedo@ulster.ac.uk (J.M.C.); 2 Department of Computing and Mathematics, Manchester Metropolitan University, Manchester M15 6BH, UK; s.djahel@mmu.ac.uk; 3 Applied Research, British Telecomm, Ipswich IP5 3RE, UK; ali.sajjad@bt.com

**Keywords:** IoT, lightweight cryptography, smart home, security, privacy preservation, data anonymisation

## Abstract

Internet of Things (IoT) technology is increasingly pervasive in all aspects of our life and its usage is anticipated to significantly increase in future Smart Cities to support their myriad of revolutionary applications. This paper introduces a new architecture that can support several IoT-enabled smart home use cases, with a specified level of security and privacy preservation. The security threats that may target such an architecture are highlighted along with the cryptographic algorithms that can prevent them. An experimental study is performed to provide more insights about the suitability of several lightweight cryptographic algorithms for use in securing the constrained IoT devices used in the proposed architecture. The obtained results showed that many modern lightweight symmetric cryptography algorithms, as CLEFIA and TRIVIUM, are optimized for hardware implementations and can consume up to 10 times more energy than the legacy techniques when they are implemented in software. Moreover, the experiments results highlight that CLEFIA significantly outperforms TRIVIUM under all of the investigated test cases, and the latter performs 100 times worse than the legacy cryptographic algorithms tested.

## 1. Introduction

The *Smart Cities* concept relies on information gathered from a myriad of tiny IoT sensors. These can be used to monitor the location or behavior of individuals as well as their health and fitness conditions, while other sensors will monitor critical infrastructure such as highways and bridges, valuable assets in Industry 4.0 and crop growth in agriculture. These sensors represent an invaluable source of information that can be efficiently processed and analyzed in an intelligent manner to improve the services offered to the cities’ inhabitants, thereby encouraging more people to adopt smart solutions and use smart devices. Since many of these services are delivered over wireless interfaces and typically carry sensitive private information, there is a need for a comprehensive approach to security and privacy-preservation. These requirements must also be balanced with the need for a particular Quality of Service (QoS) in terms of bandwidth and latency which in turn drives the need for more efficient use of costly or scarce resources such as radio network capacity and edge devices’ computational capabilities [1].

Achieving this, however, is very challenging for several reasons;

- The limited computational capabilities and the inherent design constraints of the sensors make them an easy target for hackers [2];
- The large volume of collected data may include erroneous or intentionally injected malicious data that can lead to serious effects on the system operation and integrity;
- Compliance with the General Data Protection Regulation (GDPR) introduces constraints that restrict the usage and sharing of the data collected by companies offering smart home services. Mechanisms are therrefore required for those users who are unwilling to share potentially identity-disclosing data with the service providers;
- Inter-operability between devices from different manufacturers makes it difficult to build a unified smart home system.

To this end, we propose an IoT-based smart home architecture to support various applications, discuss several security threats and potential countermeasures, and finally undertake an experimental study to identify the most suitable cryptography algorithms for use in this context. The main contributions of this paper are summarized as follows:

- Proposing a simple yet comprehensive IoT-based smart home architecture that could serve as a reference model for future works aimed at designing improved smart home systems.
- Analyzing the potential security threats that may target such an architecture and outlining the existing countermeasures.
- Designing a new security parameter selection algorithm that enables any newly added IoT device to be configured with the most suitable lightweight cryptographic algorithms.
- Conducting an experimental study to evaluate the suitability of several hardware-oriented lightweight cryptography algorithms to secure the IoT devices used in this architecture.

The remainder of the paper is organized as follows. Section 2 summarizes the literature followed by a description of the proposed IoT enabled smart home architecture in Section 3. In Section 4, we present examples of smart home applications that can be built upon this architecture, then we highlight their associated security threats in Section 5. In Section 6, we outline several legacy and lightweight cryptographic algorithms that can be used to mitigate the above threats. A new algorithm that enables selecting the most suitable cryptographic algorithms for configuration in IoT devices is proposed in Section 7, followed by an experimental evaluation of the achieved encryption/decryption time for a selection of lightweight and legacy cryptography algorithms in Section 8. Finally, we conclude in Section 9.

## 2. Related Work

In recent years, IoT technology revolutionized the world by substituting humans with intelligent devices to perform many everyday tasks [3,4]. These smart devices become responsible for handling the data as conventional cities undergo the transformation to smart cities [5], homes to smart homes [6], industry to industry 4.0 [7] and so on. Despite the numerous benefits that IoT technology brings, there is a need to ensure the security of the IoT infrastructure and the privacy of the collected data due to the inherent characteristics of the IoT framework and the rapidly expanding spectrum of cyber attacks [8,9].

Several architectures were proposed to add security and privacy features to various working scenarios of IoT networks. Hamed et al. proposed a secure Artificial Intelligence (AI)-based architecture for securing the edge layer of an IoT framework [10]. In this paper, the life cycle of any attack is detected and categorized using the Cyber Kill Chain model. The types of threats and their handling by AI engines for the edge layer were also discussed. Recently, a new safe model for IoT was proposed for supply chain risk management [11]. The designed architecture provides security by applying machine learning techniques, cryptographic hardware monitoring and distributed system coordination.

Another multi-layered scheme for secure data transportation between IoT devices connected through a cellular network was developed in [12]. This scheme provides a secure end-to-end communication system for IoT that is comprised of secure interlocking functional elements in the carrier network. A hybrid scheme based on blockchain technology was introduced for ensuring end-to-end security [13]. The proposed scheme uses the Authentication and Authorization for Constrained Environments (ACE) framework [14] for blockchain authorization and the Object Security Architecture for the Internet of Things (OSCAR) object with group key security. In [15], the authors used the concept of decentralized fog computing architecture to map privacy patterns for IoT. They used a smart vehicle use case scenario as a proof of concept to elaborate how privacy-by-design can be used in a practical instance to preserve users’ privacy. The works discussed above mostly provide a proof of concept for the proposed IoT architecture but very few of them provided working evidence of such an architecture. Given the limited resources of typical IoT systems, it is important to analyze the performance of security algorithms in real-world IoT scenarios.

There are several papers that discussed the performance of security algorithms for IoT devices when proposing a new IoT model. Recently, a survey paper published a detailed description of lightweight algorithms, but no real-time experiments were conducted for performance analysis [16]. Buchanan et al. published a paper on lightweight cryptography methods and performed an in depth analysis of some of them [17]. They used Fair Evaluation of Lightweight Cryptographic Systems (FELICS) bench-marking to test the efficiency of algorithms for software implementations on 8-bit, 16-bit, and 32-bit micro-controllers. They performed a fair comparison and obtained some interesting findings, but their experiments were conducted for fixed block sizes and no real-world use cases were evaluated using hardware implementations or IoT sensors.

A high-performance and low-energy implementation of cryptographic primitives was carried out for programmable system-on-chip IoT devices in [18]. The authors used Field Programmable Gate Arrays (FPGAs) to implement AES, Rivest-Shamir-Adleman (RSA), Data Encryption Standard (DES) and Secure Hash Algorithm (SHA) algorithms for testing and analyzing them for different performance metrics. The authors highlighted important findings, such as that the achieved performance boost and energy savings in FPGA implementations compared to software implementations range from 1.5× to 2983×, and from 1.8× to 4033×, respectively across a variety of cryptographic algorithms, but unfortunately the algorithms they used are not lightweight. FPGA boards possess powerful computing capabilities that cannot be compared with the limited computing resources of IoT sensor nodes. Peireira et al. considered a Wireless Sensor Network (WSN) scenario and performed detailed experimentation on several devices and different operating systems [19]. The work also used a small set of randomly selected cryptographic algorithms, such as AES, Curupira and Trivium, including some algorithms that were not standardized, such as Marvin [20].

To complement the above efforts, this paper introduces a new architecture that can support several IoT assisted living applications with a specified level of security and privacy preservation. This architecture is supported by a new algorithm to ensure that any newly added IoT sensor is configured with the most suitable cryptographic suite based on the device capabilities and the target security application. An experimental study is also conducted to provide time latency comparison between legacy and lightweight cryptographic algorithms when implemented on a prototype IoT network.

In this section, we will provide a detailed description of our proposed generic IoT enabled smart home architecture, highlighting the need for each of its main phases and explaining the interaction among them, as well as discussing how the data flows between the different components.

**Figure 1:**
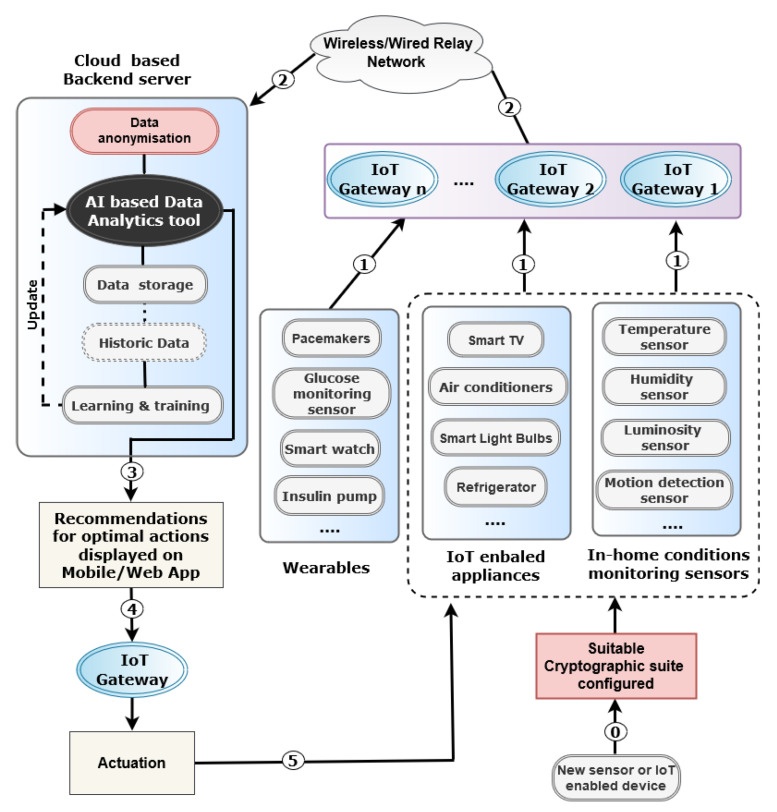
Architecture of a secure and privacy-preserving IoT-based sensing and actuation system in a smart home.

The proposed architecture is shown in Figure 1 indicating that the data are collected from various heterogeneous IoT sources, then anonymised, processed and analyzed using AI (Artificial Intelligence)-based techniques. Based on the outcome of this analysis several actions are taken to optimize some parameters of interest in order to achieve the desired security and performance objectives of the smart home application in use. To ensure a secure and privacy-preserving smart home environment with optimized usage of the available resources, e.g., electricity, water and gas, the following phases are required.

## 3. IoT Enabled Smart Home Architecture

### 3.1. Phase 0—Security Parameters Configuration

This phase is required for a new IoT device before connecting it to the smart home system. The IoT device is on-boarded with a suite of cryptographic algorithms to cater for the confidentiality, integrity and authentication requirements of the applications and systems using this specific device.

This phase plays a key role in the protection of the smart home from several security threats as explained later in Section 5.

### 3.2. Phase 1—Data Sensing and Reporting

We can distinguish three main classes of IoT devices that could be used in a smart home environment: wearables, IoT enabled appliances and in-home conditions monitoring sensors. Each device senses one or more parameters and sends the data readings either periodically or when certain conditions are met, depending on its configuration. If multiple IoT Gateways are within range, the IoT devices need to choose the most trustworthy or reputable gateway to forward their data. Here, a trust management scheme is mandatory [21].

### 3.3. Phase 2—Data Aggregation and Relaying

Once the data are received at the IoT Gateway level, it will either relay them to their destination through the relay network or store them first and then aggregate them with other readings before relaying the aggregated values [22]. The decision to aggregate or relay immediately depends on the target application as well as the data reading value. Alternative dedicated IoT Gateways could be deployed as well to operate as an edge server for heavyweight tasks that cannot be handled by the IoT devices.

Several security mechanisms could be configured at the IoT Gateway level, such as an application proxy firewall and network based IDS (Intrusion Detection System), to offload the IoT devices from this moderate to heavy processor-intensive task and ensure early detection of security threats. It is worth noting that some new generation high-end broadband routers could also operate as an IoT Gateway for several IoT devices, such as the mesh-IoT hybrid router (https://www.tp-link.com/uk/press/news/18045/) unveiled by TP-link in August 2018 and which unifies the control of smart home IoT devices.

### 3.4. Phase 3—Cloud-Based Data Analytics

A cloud-based service is usually used to store, process and analyze the aggregated data sent by the IoT Gateways. The received data will first be anonymised to preserve the privacy of the concerned individuals, depending on the target application, and ensure that any further processing will not provide any identity-disclosing information [23]. There are different techniques that can be used for this purpose such as Data Generalization and Differential Privacy which are the two techniques currently used by google to protect its customers’ data (https://policies.google.com/technologies/anonymization?hl=en).

The former technique consists of removing a portion of data or replacing some elements with a commonly used value in order to hide the identity of the concerned individuals. The latter technique adds mathematical noise to the collected data in a way that makes it difficult to ascertain whether a given individual is part of a dataset. It is worth noting that this technique may reduce the utility of the data. That is why we strongly suggest using data generalization based anonymisation in smart home applications. The main challenge when designing data generalization techniques is how to achieve data anonymisation while at the same time minimizing the information loss due to the modification of the original data. K-anonymisation techniques [24] are among the most widespread techniques used for this purpose, thus we propose their use in this architecture.

The anonymised data will then be passed to the AI-based data analytics tool [25], usually using a machine-learning algorithm, to extract useful knowledge from the processed and analyzed data so that an accurate perception of the monitored environment (smart home and its occupants in this case) is formed. This perception will result in a set of optimal actions/adjustments to be proposed for the actuation system in place. Subsequently, the processed data will be stored and become historic data that will be used for enhancing the learning and training of the machine-learning model so that updates will be applied to the data analytics tool to achieve higher accuracy.

### 3.5. Phase 4—Optimal Decisions Delivery

In this phase, the optimal actions nominated in the previous phase will be communicated to the actuation system through the IoT Gateway. Alternatively, they could be also displayed to the users through a mobile or web app so that they can update the settings accordingly or just for information purposes.

### 3.6. Phase 5—Actuation

Finally, upon receiving the above mentioned nominations, the actuation system will instruct the concerned devices to make the requested adjustments through sending an updated configuration file, for example. The actuation process ranges from changing the upper and lower limits of a given parameter so that a reading beyond these limits should trigger an alert, to updating the frequency at which different parameters are measured or the conditions under which certain tasks are performed.

## 4. Potential Use Cases

In a smart home context, several heterogeneous resource-constrained IoT sensors and actuators as well as IoT enabled appliances are usually used to provide data inputs to a Smart Home app to enable the monitoring and adjustment of certain parameters. The Smart Home app runs software that uses these data inputs along with AI-based techniques, such as machine learning and deep learning algorithms [25], to build a perception about the home physical environment and its occupants. This perception will drive the decisions made by the Smart Home app, as explained in Section 3.4, about the actions to be initiated in order to achieve the desired objectives such as optimizing the energy-consumption, monitoring the physical activities or behavior of the home residents (e.g., children and elderly), etc.

Several applications could emerge from this scenario such as:

- **Automation of energy consumption optimization** of different smart appliances (heaters, lights, Smart TV, entertainment devices, etc.). Assume that in the living room the sofa and chairs are equipped with embedded sensors to detect the presence of a human; this data will be correlated with the Smart TV control unit so that it is switched off (or put on standby mode) whenever human presence is not detected in the living room for a given period (e.g., *x* minutes).
- **Occupancy detection** inside a room, house or a building for either energy usage optimization or for detection of any unwanted entry to properties.
- **Activity or abnormal events recognition** to detect specific events of interest such as flood, fire etc., or for monitoring the activities of elderly or people with chronic illness at home. In the case of any unusual activity or out-of-range readings (i.e., a value that exceeds a certain threshold) an alert is triggered and the concerned individuals/services are notified.
- **Continuous health monitoring** for residents using either on-body or off-body sensors and reporting the measured data through an IoT Gateway to remote healthcare service.

The above applications could be an easy target for security attacks, in particular if multi-hop transmission is used, with consequences varying from disturbing the optimal operation of the system in place to more severe ones. That is why the authentication of the sender device is required as well as the verification of the authenticity of the data reported to prevent false alerts. Mechanisms to prevent the modification of such data in transit are also required. Despite the multiple advantages that IoT brings to individuals living in smart homes, their privacy might be compromised if weaknesses related to IoT devices configuration (e.g., devices deployed with their default passwords or the latest security updates are not installed) are discovered and exploited by hackers. According to a recent article published by MIT Technology Review (https://www.technologyreview.com/f/614062/russian-hackers-fancy-bear-strontium-infiltrate-iot-networks-microsoft-report), a group of hackers associated with Russian spy agencies were using IoT devices to break into corporate networks. This shows how important is the security of these devices and the extent of the damage that can be caused in case of a successful attack.

## 5. Potential Security Threats

In this section, we will discuss several security threats that can target our proposed IoT enabled smart home architecture.

The success and wide adoption of secure IoT architectures and the different applications that they can support is reliant upon gaining the trust of their potential users [26]. Such trust is very important due to the harm caused to the security of an individual’s physical, financial and social life if their personal information is stolen or misused. Therefore, ensuring that adequate security measures are implemented to tackle potential security threats is paramount. Below, we briefly discuss several security threats.

### 5.1. Corrupted or Forged Data

This serious attack can disturb the proper operation of the deployed monitoring system, as decisions made based on incorrect data will not achieve the desired IoT system objectives such as energy consumption reduction, etc. The root causes of this attack are the following:

- Deliberate malicious mis-configuration of IoT devices, leading to erroneous data generation. For example, increasing all reported power consumption.
- Compromised IoT devices generating forged data readings. For example, reporting power consumption to be a randomly generated value.
- Data modification in transit, in multi-hop communication, by a compromised IoT Gateway.

### 5.2. Replay Attacks

This attack can happen if the IoT devices generating data do not implement an anti-replay scheme, such as adding a protected timestamp to the data packets, and may lead to outdated data being used and incorrect decisions being made based on it.

### 5.3. IP Spoofing and Identity Usurpation

Without a sophisticated data-origin authentication mechanism, such as IPsec, identity usurpation and IP spoofing attacks can easily target IoT devices using other devices connected to the same network. A rogue IoT device connected to the smart home network can launch an IP scanning attack to discover the available IP addresses and then use them to mount more sophisticated attacks such as data manipulation or Denial of Service (DoS) or DDoS (Distributed DoS) attacks, as explained below.

### 5.4. DoS/DDoS Family Attacks

Any security threat that can result in a service or a resource being unavailable can be qualified as a DoS attack. In this architecture, any IoT device, if not adequately protected, could be infected by a Trojan horse, which is a malicious code remotely controlled by an attacker, either installed by having physical access to the device or through remote updates. Accessing such a device could be the first step towards launching a successful DoS or DDoS (Distributed DoS) attack. These IoT devices can be exposed to several types of Botnets with varying consequences for the efficiency of the monitoring system and also the availability of the provided services. A recent Blockhain based IoT infrastructure proposed in [27] could offer a resistant solution to DDoS.

### 5.5. Data Leakage

In the architecture shown in Figure 1, if a hacker successfully gains access to the database of the cloud-based backend server, where historic data are stored, private and potentially sensitive information about the smart home occupants could be disclosed if robust anonymisation schemes and access control mechanisms are not implemented.

## 6. Potential Countermeasures: Lightweight vs. Legacy

In this section, we will outline several legacy and lightweight cryptographic algorithms that can be used to encrypt/decrypt the data collected or aggregated by individual IoT devices so that one or more of the attacks outlined above can be mitigated.

Designing efficient countermeasures to one or more of the above threats is a challenging task due to the stringent constraints of the devices on which the solution will be implemented and operate to provide the desired level of protection. Several legacy and lightweight cryptographic algorithms exist for this purpose but their suitability for use in different IoT devices vary significantly. For this study we selected the algorithms outlined below and we evaluated and compared their performance using an experimental testbed to find out which algorithms could be used to setup and configure cryptographic suites on new IoT devices before they are integrated into the smart home system (see Section 3.1).

For legacy cryptographic algorithms we consider the following:

- Block Ciphers: Blowfish [28], AES128-CBC and AES256-CBC [29].
- Stream Ciphers: Chacha20 [30], AES128-CTR, AES256-CTR and DES3 [31].

For lightweight cryptographic algorithms we selected CLEFIA and Trivium as they are part of the ISO/IEC 29192-3:2012 Lightweight Cryptography Standard, as well as being suitable for the experimental use case of this study.

CLEFIA [32]: A 128-bit block cipher (supporting 128-bit, 192-bit, and 256-bit keys) designed with the aim of achieving a good trade-off between three fundamental metrics for practical ciphers: *(i)* the achieved security level, *(ii)* the operation speed, and *(iii)* the implementation cost. Several design aspects were taken into consideration to ensure its efficient implementation in both hardware and software. CLEFIA’s immunity against several known attacks that use different techniques to recover the encryption key was proven and thus it can be used to protect data sent by IoT devices from data modification attack discussed in Section 5.1.

TRIVIUM [33]: This is a hardware-oriented binary additive stream cipher that is considered both secure and efficient. It was designed to explore the possibility of simplifying a stream cipher without reducing its security. It is considered a compact algorithm suitable for environments with restricted gate count. It is therefore designed to be energy-efficient so that it can be implemented on tiny devices with limited power resources, and fast enough to accommodate the needs of applications requiring high-speed encryption.

## 7. Security Parameters Selection Algorithm

In order to maintain the desired security and privacy preservation level of the architecture shown in Figure 1, every newly added IoT device needs to be configured with the most suitable lightweight cryptographic algorithms required to enable security services to run efficiently. To this end, we designed a security parameter selection algorithm. This algorithm processes the system capabilities and requirements (the inputs) and provides one or more suggested actions (the outputs).

- **Inputs**: the device capabilities (e.g., CPU, RAM etc.) and the security objectives that usually depend on the target application (e.g., authentication, confidentiality, Anti-DoS protection, etc.).
- **Knowledge base**: A list of constraints or minimum operational requirements of each available lightweight cryptographic algorithm (i.e., minimum memory, minimum CPU speed, energy consumption etc.).
- **Output**: the most suitable cryptographic suite (i.e., the list of cryptographic algorithms that need to be configured in the newly introduced IoT device in the smart home monitoring system). If more than one algorithm are suggested then their usage will be in the order on which they appear.

The main steps of the proposed algorithm are described as follows.

- **Step 1—Loading the device capabilities**: A configuration file containing the new device’s capability information is loaded. This file contains the following information: CPU speed, RAM capacity, storage capacity and battery capacity.
- **Step 2—Extracting information from the knowledge base**: From the knowledge base, we create a list of algorithms with their corresponding requirements to run efficiently on IoT devices (i.e., the algorithm can run fast and does not lead to quick depletion of the device energy resources).
- **Step 3—Selecting the most suitable algorithm**: In this step, Algorithm 1 is executed and as a result the most suitable cryptographic suite is returned. The variables used in this algorithm are explained below. Algo: one of the available cryptographic algorithms.

$Algo_{Req}$: the list of requirements of the algorithm as extracted from the Knowledge base.

$Algo_{Sec}$: the list of security objectives that this algorithm can achieve, e.g., confidentiality (through encryption) only, or confidentiality and authentication, or authentication only, etc.

$List_{Algo}$: the list of available algorithms.

$List_{Cand}$: the list of algorithms for which the IoT device meets their requirements.

$Chosen_{Algo}$: the most suitable algorithm(s) among those in $List_{Cand}$.

$Security_{Serv}$: the list of all possible security services needed for different Smart Home applications, e.g., authentication, confidentiality, integrity, DoS protection, etc.

Please note that an IoT device meets all the requirements of an algorithm in the list $List_{Algo}$ if and only if the following is true: “device CPU speed ≥ min CPU speed AND device memory ≥ min memory capacity AND device battery capacity ≥ min battery capacity”. Moreover, reordering the $Chosen_{Algo}$ list means that the algorithm that satisfies most of the security objectives will be recommended first, and so on. This, of course, applies in cases where we have more than one algorithm in the list.
**Algorithm 1:** Algorithm operations
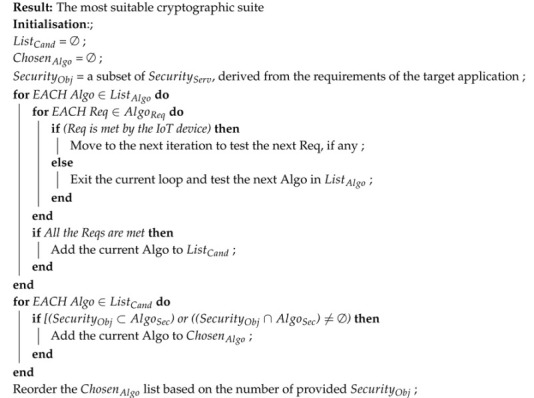


## 8. Experiments

As discussed earlier in Section 3, any new IoT device should be configured with a suitable cryptographic suite of algorithms, before connecting it to the smart home architecture, in order to ensure that the applications and services using this device can experience optimal security and performance. The purpose of the experiments developed in this study is, therefore, to identify which cryptographic algorithms, among the ones discussed in the previous section, could potentially be configured for use by the IoT devices of our proposed architecture. This is the first step in our efforts to create a prototype of a real smart home environment where numerous interesting applications could be tested.

In these experiments, we used open source software, off-the-shelf hardware and default configurations for all systems, unless otherwise detailed below.

### 8.1. Testbed Overview

**Figure 2:**
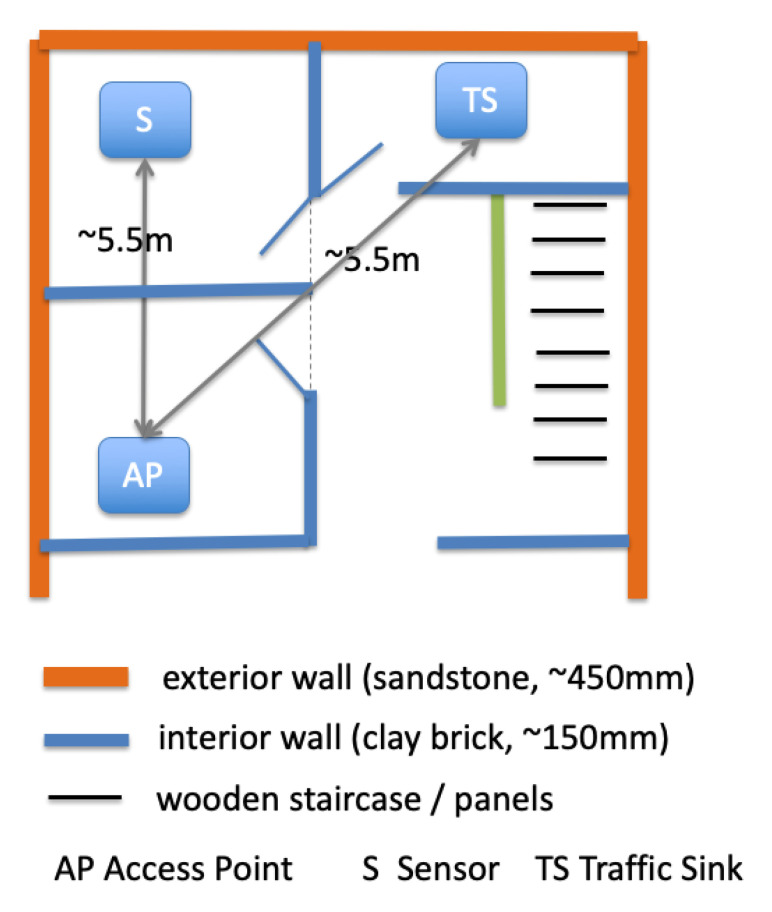
Schematic of the testbed showing physical connectivity.

**Figure 3:**
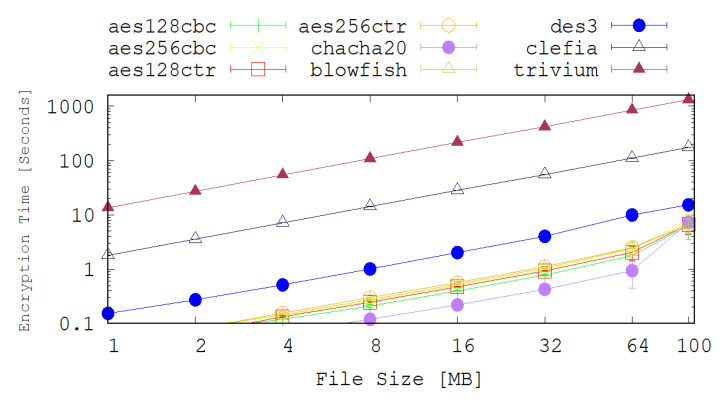
A comparison of the achieved encryption time (in ms) by several legacy and lightweight cryptography algorithms.

**Figure 4:**
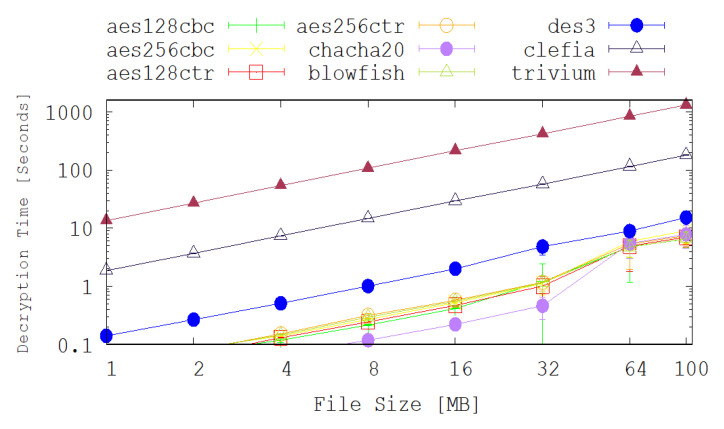
A comparison of the achieved decryption time (in ms) by several legacy and lightweight cryptography algorithms.

The testbed, shown in Figure 2, consists of a single sensor kit (Raspberry Pi 3), with another host operating as a traffic sink (a MacBook Pro) and a wireless access-point (TP-Link) to provide wireless network connectivity between the two peers. The experiments were set up in a smart home environment which is the home of one of the authors. The scenario evaluated consists of encrypting/decrypting files on the sensor *S* and then sending them to the traffic sink *TS* via the Access Point *AP*. The size of files sent by the sensor *S* were set to 1, 2, 4, 8, 16, 32, 64 and 100 MB, and this experiment was repeated 50 times for each of the nine cryptographic algorithms evaluated. We used an NTP (Network Time Protocol) local time server to synchronise the sensor and sink clocks in order to achieve precise time stamping. The metrics measured are the encryption and decryption times at the sensor side and the results shown in Figure 3 and Figure 4 are the average values of 50 experiments.

### 8.2. Results Analysis

The achieved encryption and decryption times by each of the selected legacy and lightweight cryptographic algorithms are depicted on the graphs shown in Figure 3 and Figure 4. Apart from the Chacha20 and DES3 algorithms, most of the chosen legacy cryptography algorithms have very similar performance results in terms of the encryption and decryption times. The encryption and decryption time of DES3 is 2 times slower than the other algorithms in most of the cases. For example, for 100 MB files scenario, the average encryption time for DES3 is 15.3 s while for AES128-CTR it is 6.62 s. On the other hand Chacha20 performs better than any of the chosen cryptography algorithms up to 32MB file size. In the case of 32 MB file scenario, the average encryption time for Chach20 is 0.42 s while the AES128-CTR is 0.92 s.

Figure 3 and Figure 4 also show the achieved encryption and decryption times for several the lightweight cryptography algorithms. These figures reveal that CLEFIA significantly outperforms TRIVIUM under all of the test cases explored here. It is also noted that, counter-intuitively, the encryption and decryption times for the lightweight cryptography algorithms are considerably higher than their legacy counter-parts. This is due to how these new techniques are intended to be deployed. For example, TRIVIUM is designed for use in a hardware solution and is optimised to reduce the number of gates that are required to achieve such an implementation. In our tests, however, the algorithms are implemented in software and are clearly performing poorly relative to the legacy techniques. Since TRIVIUM takes more than 100 times longer to encrypt a file, in this software implementation, it follows that it will consume approximately 100 times more energy and that will have an associated reduction in battery lifetime and increase in latency.

The results also show that CLEFIA performs approximately 10 times slower than the legacy implementations. This tends to suggest that software implementations of this algorithm in a high level language such as Python are not suitable for IoT applications. It may be probable that low level, machine code implementations and hardware implementations will perform better.

## 9. Conclusions

An IoT enabled smart home architecture was proposed in this paper to support several secure and privacy preserving applications in smart cities. Due to the large variety and heterogeneity of IoT devices and the security objectives of the applications using them, every new IoT device is configured with a suite of lightweight cryptographic algorithms before integrating it to the system. An experimental study was conducted to evaluate several legacy encryption/decryption techniques and compare them with more recently proposed lightweight techniques. The results clearly show that the hardware-oriented lightweight techniques perform significantly worse than the legacy techniques when they are implemented in software. The software implementation of CLEFIA, for example, in Python leads to a significant reduction in its performance as the results highlight that it performs 10 times slower than the legacy algorithms. Developers of IoT security systems therefore need to be mindful of the type of platform that a candidate encryption/decryption technique was developed for.
